# Ancient Pbx-Hox signatures define hundreds of vertebrate developmental enhancers

**DOI:** 10.1186/1471-2164-12-637

**Published:** 2011-12-30

**Authors:** Hugo J Parker, Paul Piccinelli, Tatjana Sauka-Spengler, Marianne Bronner, Greg Elgar

**Affiliations:** 1 Division of Systems Biology, MRC National Institute for Medical Research, The Ridgeway, Mill Hill, London NW7 1AA, UK; 2 Weatherall Institute of Molecular Medicine, University of Oxford, John Radcliffe Hospital, Oxford OX3 9DS, UK; 3 Division of Biology, California Institute of Technology, Pasadena, CA 91125, USA

**Keywords:** Gene regulation, enhancer code, sea lamprey, Hox genes, embryogenesis

## Abstract

**Background:**

Gene regulation through *cis*-regulatory elements plays a crucial role in development and disease. A major aim of the post-genomic era is to be able to read the function of *cis*-regulatory elements through scrutiny of their DNA sequence. Whilst comparative genomics approaches have identified thousands of putative regulatory elements, our knowledge of their mechanism of action is poor and very little progress has been made in systematically de-coding them.

**Results:**

Here, we identify ancient functional signatures within vertebrate conserved non-coding elements (CNEs) through a combination of phylogenetic footprinting and functional assay, using genomic sequence from the sea lamprey as a reference. We uncover a striking enrichment within vertebrate CNEs for conserved binding-site motifs of the Pbx-Hox hetero-dimer. We further show that these predict reporter gene expression in a segment specific manner in the hindbrain and pharyngeal arches during zebrafish development.

**Conclusions:**

These findings evoke an evolutionary scenario in which many CNEs evolved early in the vertebrate lineage to co-ordinate Hox-dependent gene-regulatory interactions that pattern the vertebrate head. In a broader context, our evolutionary analyses reveal that CNEs are composed of tightly linked transcription-factor binding-sites (TFBSs), which can be systematically identified through phylogenetic footprinting approaches. By placing a large number of ancient vertebrate CNEs into a developmental context, our findings promise to have a significant impact on efforts toward de-coding gene-regulatory elements that underlie vertebrate development, and will facilitate building general models of regulatory element evolution.

## Background

*Cis*-regulatory elements play an essential role in the precise co-ordination of vertebrate development as illustrated by the increasing number of examples where mutations in such sequences lead to developmental malformations [1-3]. One of the major challenges in modern biology is the deciphering of the regulatory language, syntax and grammar, encoded in the genome, that directs spatio-temporally restricted gene expression. To achieve this requires the identification and functional characterisation of *cis*-regulatory elements, followed by the deconvolution of the TFBSs therein.

*Cis*-regulatory elements can be predicted by sequence conservation analysis, as tight clusters of functional TFBSs can be under strong evolutionary constraint [4-7]. Alternatively, targeted approaches involving chromatin immunoprecipitation (ChIP) can be used to identify binding-events between specific transcription factors and DNA [8-10]. These are complementary approaches, as ChIP analyses are restricted to identifying regulatory regions that are targets of selected TFs at the particular time-points chosen for the analysis, whilst sequence conservation can identify elements irrespective of the TFs that bind to them or the developmental time-points at which they act. Sequence conservation can also provide evidence for ancient gene regulatory network (GRN) interactions that are shared between species. Comparative approaches applied to vertebrate genomes have identified a set of putative regulatory elements showing extreme conservation across mammals (Ultra-conserved elements [11]), as well as Conserved Non-coding Elements (CNEs) shared between mammals and fishes [6,12,13]. These elements are clustered around developmental genes [6,12] and a large proportion of CNEs that have been tested in transgenic assays drive spatially restricted reporter gene expression in mouse or zebrafish embryos [6,7,14]. Furthermore, a number of CNEs have been shown to have roles in developmental diseases [1,15]. Despite their high sequence conservation between vertebrates, only a minute fraction of CNEs can be traced back to invertebrate chordates [16]. Thus, CNEs represent a set of *cis*-regulatory elements that are likely to be fundamental during development of the vertebrate body plan and comprise a valuable resource for deciphering the genomic regulatory code for vertebrate development.

Phylogenetic footprinting has been successfully implemented to identify TFBSs that play key roles in the action of individual CNEs [17-19]. However, there have been very few studies seeking to place large numbers of deeply conserved CNEs into a developmental context through using this approach [20,21]. Furthermore, despite progress having been made identifying key sequence motifs within vertebrate promoter elements [22], ancient CNEs have remained somewhat recalcitrant to systematic motif-identification algorithms, despite some elegant targeted approaches. Within non-coding elements conserved amongst mammals, a large number of long motifs [12-22 nucleotides) [23] and some shorter motifs [24] have been identified as overrepresented. However, the majority of these were not matched to any known factors, nor linked to any patterns of enhancer activity, so the biological significance of these motifs is hard to interpret. Recently, an elegant study used a classifier algorithm to identify sequence motifs predictive of heart enhancer activity in mammalian CNEs [25]. However, mammalian CNEs represent a set of sequences that only partially overlap with the more ancient mammal-fish CNEs and it is not clear to what extent they are functionally and mechanistically alike. Studies seeking to identify motifs that contribute to tissue-specific expression of deeply conserved vertebrate CNEs have discovered novel motifs associated with forebrain enhancer activity [7,21]. As part of a large-scale project to characterise the *in-vivo *enhancer activity of CNEs, Pennacchio *et al*. [7] used four human-fugu CNEs that drove forebrain reporter expression in mouse embryos to identify 6 enriched 5 bp-long sequence motifs. 23 elements enriched for these motifs were tested for enhancer activity, of which 4 were found to drive forebrain expression - an enrichment for this expression domain compared to the original enhancer set. Li *et al*. [21] characterised 13 CNEs driving forebrain reporter expression in zebrafish embryos, identifying 5 enriched motifs of 6 bp and demonstrating that these sequences contributed to forebrain enhancer activity. These investigations go some way towards providing a developmental context for the CNEs with those motifs, but this is somewhat limited by the factors that bind to them remaining uncharacterised. Whilst it is unclear to what extent ancient vertebrate CNEs are composed of 'conventional', previously characterised TFBSs, candidate motif search approaches have provided evidence that mammalian UCEs are enriched for known TFBS motifs [26] and that ancient vertebrate CNEs associated with genes involved in CNS development show enrichment for Oct and Sox motifs [20]. The success of these isolated studies hints that it may be possible to systematically identify functional TFBSs within CNEs by phylogenetic footprinting.

The sea lamprey (*Petromyzon marinus*) hails from an anciently diverging jawless vertebrate lineage, the agnathans, which split from the jawed vertebrate lineage 550-650 million years ago [27]. We have previously found a significant number of CNEs that are conserved between lamprey and jawed vertebrates [28]. We predicted that the relatively low sequence identity between the lamprey and jawed-vertebrate homologous elements would facilitate the identification of conserved TFBS motifs within them. In addition, characterisation of these motifs could illuminate ancient GRN interactions common to all vertebrates. Thus, we sought to identify TFBS motifs in CNEs by performing phylogenetic footprinting, using the lamprey elements as a guide.

Here we identify deeply conserved TFBS motifs for the Pbx-Hox heterodimer within a cluster of CNEs associated with the *meis2 *gene. We use *in-silico *analyses to demonstrate that jawed vertebrate CNEs and other sets of conserved vertebrate enhancers are highly enriched in Pbx-Hox motifs. Using reporter assays in zebrafish and lamprey embryos, we show that these motifs correlate with enhancer function in the hindbrain and pharyngeal arches. These results represent a further step toward de-coding vertebrate CNEs, allowing a large proportion of them to be more firmly placed into a developmental context and revealing ancient gene regulatory network interactions for hindbrain patterning that are shared across vertebrates. Finally, our findings enable us to hypothesise an evolutionary scenario regarding the role of many CNEs in the evolution of the vertebrate hindbrain and the branchial region of the head.

## Results

### A set of *meis2 *CNEs drive expression in the hindbrain and cranial ganglia in zebrafish and lamprey embryos

**Figure 1:**
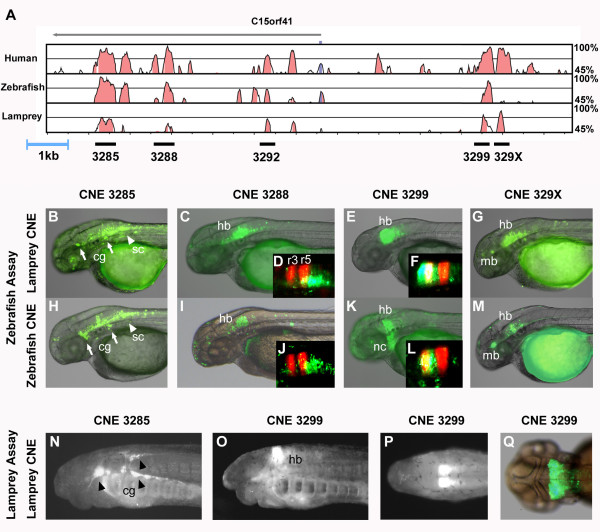
**Meis2 CNEs from zebrafish and lamprey drive equivalent expression patterns in zebrafish and lamprey embryos**. **A**, multiple alignment of orthologous genomic regions containing the gene *c15orf41 *(blue peak), downstream of *meis2*, revealing CNEs (red peaks). Human, zebrafish and lamprey sequences are aligned with the fugu sequence as a baseline. Zebrafish CNE 329X is translocated in the current zebrafish genome assembly so does not appear in this alignment. **B-M**, orthologous elements from lamprey (**B-G**) and zebrafish (**H-M**) drive similar GFP expression patterns in the nervous system of zebrafish embryos at 54hpf: element 3285 in the cranial ganglia (arrows) and primary neurons of the hindbrain and spinal cord (arrowhead) (**B, H**); 3288 in neurons of the hindbrain posterior to rhombomere (r) 4 (**C, I**), as determined by comparison with r3r5 RFP expression (**D, J**); 3299 in the anterior hindbrain - r2-4 for the lamprey homolog (**E, F**) and r3-4 plus the corresponding neural crest for the zebrafish homolog (**K, L**); 329X in the hindbrain and neurons of the midbrain (**G, M**). **N-O **, embryonic day 14-15 lamprey embryos transgenic for lamprey elements 3285 (**N**) and 3299 (**O**) show GFP expression in the cranial ganglia (arrowheads) and anterior hindbrain respectively, consistent with their expression in zebrafish (3285: **B**, 3299: **E**). **P-Q**, dorsal views of the head of lamprey (**P**) and zebrafish (**Q**) embryos transgenic for lamprey element 3299. cg: cranial ganglia; hb: hindbrain; mb: midbrain; nc: neural crest; sc: spinal cord.

We previously identified a genomic region, downstream of the developmental gene *meis2*, containing a number of CNEs that are conserved between jawed vertebrates and lamprey [28] (Additional File 1). We grouped these CNEs into five separate elements (Additional File 2) for functional testing in a zebrafish tol2 reporter assay [29]. Four of these elements drive discreet and complementary patterns of reporter expression in the hindbrain of zebrafish embryos, with homologous zebrafish and lamprey elements driving highly similar expression patterns (Figure 1). These patterns of reporter expression are consistent with the endogenous expression of *meis2 *in the hindbrain [30,31], where Meis proteins play a crucial patterning role by interacting with Hox and Pbx transcription factors [32,33]. Lamprey and zebrafish CNE 3285 elements both drive GFP expression in the cranial ganglia and primary neurons of the hindbrain and spinal cord. CNE 3288 elements of zebrafish and lamprey drive GFP in neurons of the hindbrain posterior to rhombomere 4 (r4), as shown by comparison to RFP expression in r3 and r5 in a transgenic line containing RFP under the control of a *krox20 *regulatory element [34]. CNE 3299 elements up-regulate GFP in the anterior hindbrain - r2-4 for the lamprey homolog and r3-4 plus neural crest migrating into the hyoid pharyngeal arch for the zebrafish homolog. CNE 329X of lamprey and zebrafish both drive GFP expression in the anterior hindbrain and neurons of the midbrain.

We have developed a parallel reporter assay in lamprey embryos (in submission) to assess the functional conservation of CNEs across vertebrates. Using this assay, we have tested lamprey CNEs 3285 and 3299 for enhancer activity during lamprey embryogenesis. In lamprey embryos, CNE 3285 drives GFP expression in the cranial ganglia and CNE 3299 in the anterior hindbrain (Figure 1). Thus, for both of these elements, the pattern of reporter expression driven in lamprey embryos is almost identical to the pattern driven in zebrafish embryos (Figure 1). This provides compelling evidence that these CNEs are part of a gene-regulatory network for hindbrain patterning that is conserved across all vertebrates.

### Some *meis2 *CNEs contain deeply conserved Pbx-Hox TFBS motifs

Because of its clear and specific expression pattern in the hindbrain of zebrafish and lamprey embryos, we chose element 3299 as a starting point for the identification of putative transcription-factor binding-sites by phylogenetic footprinting. A number of studies have documented a role for the anterior Hox proteins in regulating rhombomere-specific gene expression by binding as hetero-dimers and -trimers with the TALE-class homeodomain proteins Pbx and Meis [17,18,35,36]. These complexes bind to characteristic binding-sites composed of partially overlapping Pbx-Hox half-sites, frequently in conjunction with a distal Meis/Prep site [17,18,35,36]. In some cases it has been shown that the pbx-hox motif is both necessary and sufficient for highly specific patterns of reporter expression, for instance for activity of a mouse *hoxb1 *enhancer in r4 in the mouse hindbrain [17] and for r4 and pharyngeal arch activity of a mouse *hoxb2 *enhancer [36].

**Figure 2:**
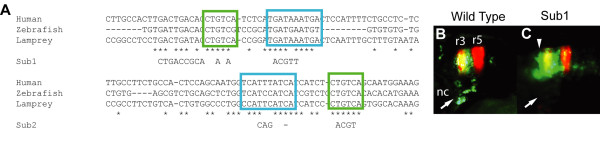
**CNE 3299 harbours essential conserved Pbx-Hox and Meis binding-sites**. **A**, multiple sequence alignment of a region of CNE 3299 from human, zebrafish and lamprey highlighting conserved Pbx-Hox (blue box) and Meis (green box) binding-site motifs. The specific sites mutated in elements sub1 and sub2 are indicated below the alignment. **B-C**, compared to wild-type 3299 expression (**B**), mutating the first Pbx-Hox and Meis motif cluster (sub1) results in the loss of reporter expression in the neural crest (arrow) and broader expression in the hindbrain (arrowhead) (**C**). nc: neural crest.

We identified two Pbx-Hox motifs within CNE 3299, conforming to the TGATNNAT consensus [37,38], that are conserved across all sequenced vertebrate genomes, each closely associated with conserved Meis motifs (TGACAG/A) [39] (Figure 2). In the zebrafish sequence, the first pair of Pbx-Hox and Meis motifs is also preceded by a Pbx-Meis motif (TGATTGACAG/A) [39]. We verified the essential nature of these motifs for rhombomere-specific activity of the enhancer through mutagenesis of the zebrafish element followed by reporter assay (Methods). Mutating the first cluster of motifs (sub1) resulted in a loss of the neural crest expression of the wild type enhancer and less anteriorly restricted expression in the hindbrain compared to the wild type element (Figure 2b, c). Mutation of the second Pbx-Hox and Meis motif cluster abrogated reporter expression by this enhancer altogether, whilst a construct in which both motif clusters were mutated (sub12) also drove no GFP expression. Interestingly, CNEs 3285, 3288 and 329X were also found to harbour conserved Pbx-Hox and Meis motifs. Together, the expression patterns of these elements in the hindbrain (3285, 3288, 3299, 329X), as well as in the pharyngeal arch neural crest (3299), suggests that these motifs may represent a common feature of CNEs that drive segment-specific expression patterns in the vertebrate hindbrain and pharyngeal arches.

### Pbx-Hox motifs are enriched in CNEs and in other sets of conserved vertebrate enhancers

In order to address how widespread Pbx-Hox motifs are across conserved vertebrate enhancers, we performed a systematic scan for these motifs in vertebrate CNEs. We searched for instances of the canonical Pbx-Hox motif, TGATNNAT, that are completely conserved across CNE multiple sequence alignments. In a set of 246 alignments of CNEs between human, zebrafish, fugu and lamprey (Methods), we identified 61 conserved motifs, representing 22 fold enrichment over shuffled alignments (Methods and Additional File 3). Furthermore, in a set of 4259 gnathostome CNE alignments (of human, fugu and zebrafish sequences), 712 conserved motifs were identified; a 9 fold enrichment compared to shuffled alignment controls.

**Figure 3:**
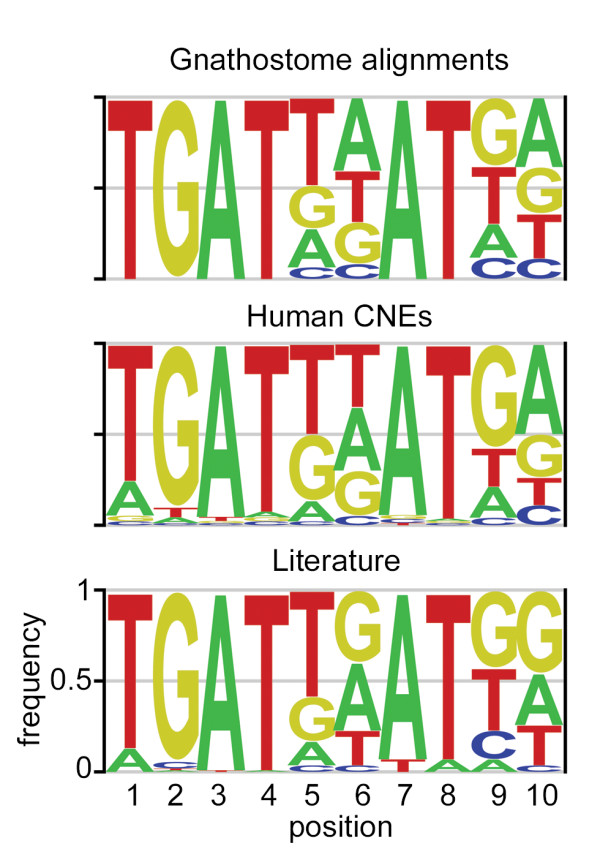
**Pbx-hox motifs in CNEs strongly resemble verified PBX-HOX binding-sites**. Position frequency logos generated from Gnathostome alignments (based on 712 conserved human TGATNNAT motifs in 4529 CNE alignments), Human CNEs (generated from the CONDOR CNE set using Cis-finder [41]) and from previous studies [36] (Literature). The relative base frequencies at positions 5 and 6, and 9 and 10, in CNEs, are in good agreement with known functional Pbx-Hox binding sites, supporting a strong KR consensus.

Further analysis of Pbx-Hox motifs in the gnathostome set reveals a paucity of cytosines at variable positions 5 and 6 (Figure 3). This is a feature of characterised Pbx-Hox binding-sites, where T, A or G at these positions contribute to determining the Hox specificity of the binding site [38,40,41]. Furthermore, positions 9 and 10, immediately 3' to the canonical Pbx-Hox motif, show strong bias towards G/T and A/G respectively, thereby defining a more stringent TGATNNATKR (KR) consensus motif that is also consistent with previously characterised Pbx-Hox binding-sites [17,18,37,38] (Figure 3). Further analysis of the lamprey and gnathostome CNE alignment sets results in even stronger enrichment for this 'KR' motif (Additional File 3).

We complemented our 'bottom-up' search for Pbx-Hox motifs in CNEs with a 'top-down' *de novo *motif search using the tool Cis-Finder [42]. Strikingly, one of the top-scoring predicted motifs identified by Cis-Finder matches our consensus KR motif for a set of 6, 693 human sequences from the CONDOR CNE database [43] ('CONDOR CNEs') (Figure 3, Methods). The KR motif occurs 562 times in this CNE set, representing a highly significant enrichment over shuffled versions of the motif (p = 5.7 × 10^-5^), and when compared to control genomic regions and the entire human genome (Table 1, Methods and Additional File 4). Interestingly, the Meis motif is also significantly enriched in the CONDOR CNE set (p = 1.0 × 10^-4^)(Additional File 5). We then examined the distribution of KR motifs in other sets of evolutionarily conserved non-coding sequences. The VISTA Enhancer Browser (EB) [44] contains over 1300 human sequences, around half of which drive reporter gene expression in mouse embryos at day 11.5. There is a significant enrichment for the KR motif (p = 0.0033) across the entire dataset compared with shuffled versions despite the fact that some of the sequences in EB are not deeply conserved (Table 1). Finally, we analysed a large set of deeply conserved human CNEs identified through comparison with the cartilaginous chimera, *Callorhinchus milii *[13], and once again found significant enrichment for the KR motif (p = 6.2 × 10^-5^) (Table 1 - 'Shark CNEs').

**Table 1:** Frequency of KR motifs, compared to shuffled versions, in different test sets

Motif	**CONDOR CNEs (from **[57]**)**	Human:Shark CNEs (from 13]	**VISTA EB (all) **[43]	**VISTA EB set HB/BA/CN+ve (from **[43]**)**	**VISTA EB set HB +ve (from **[43]**)**	**Zebrafish CBset all **[21]	**Zebrafish CB set HB +ve (from **[21]**)**
**TGATNNATKR**	**562**	**666**	**609**	**161**	**131**	**17**	**7**
TGTANNATKR	171	188	388	65	52	12	3
GTATNNATKR	150	168	279	54	39	9	2
GTTANNATKR	150	178	325	79	65	8	2
TTGANNATKR	200	245	447	80	64	9	1
TTAGNNATKR	167	238	398	74	55	7	0
ATGTNNATKR	259	297	452	86	72	20	1
ATTGNNATKR	233	297	436	74	61	20	2
AGTTNNATKR	215	254	431	85	68	9	0
TAGTNNATKR	147	154	297	54	42	10	4
TATGNNATKR	176	198	365	74	60	10	3
GATTNNATKR	274	315	419	97	74	11	0
TGATNNTAKR	106	143	314	65	50	6	1
TGTANNTAKR	142	151	421	82	60	14	1
GTATNNTAKR	59	73	195	41	34	1	0
GTTANNTAKR	105	108	253	50	33	5	0
TTGANNTAKR	162	205	385	72	62	10	0
TTAGNNTAKR	73	97	235	41	31	0	0
ATGTNNTAKR	103	124	376	64	55	3	0
ATTGNNTAKR	136	158	305	57	42	6	1
AGTTNNTAKR	85	121	320	64	50	5	1
TAGTNNTAKR	66	69	198	37	27	1	0
TATGNNTAKR	84	94	345	80	62	5	1
GATTNNTAKR	144	177	292	58	42	2	0
**mean**	165.38	196.58	353.54	70.58	55.46	8.33	1.25
**S.D**	102.75	122.06	93.73	24.74	20.92	5.52	1.67
**z-score for pbxhox**	**3.86**	**3.84**	**2.72**	**3.65**	**3.61**	1.57	**3.43**
**p-value**	5.68E-05	6.16E-05	3.30E-03	1.00E-04	2.00E-04	N/S	3.00E-04

Enrichment analysis for Pbx-Hox KR motifs, relative to shuffled versions (retaining G+C content for each binding site), within different sets of CNEs. CNEs from the VISTA enhancer browser (EB) and zebrafish cneBrowser (CB) sets have also been grouped according to annotated expression in the hindbrain (HB), branchial arches (BA) or cranial nerves (CN). All sequences are human except the Zebrafish cneBrowser set. N/S = not significant

### Pbx-Hox motifs are associated with hindbrain and pharyngeal arch CNE enhancer function

**Figure 4:**
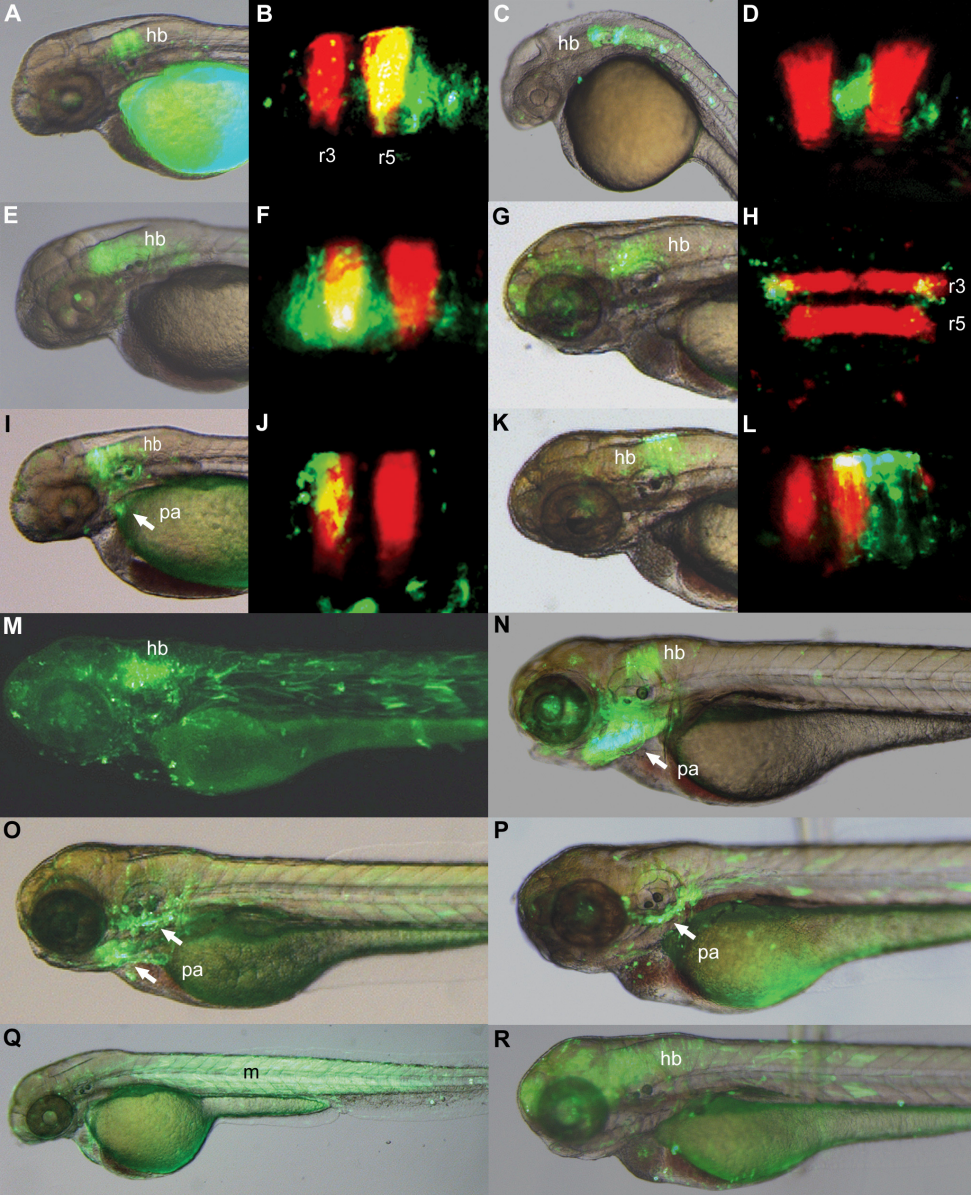
**Pbx-Hox motifs correlate with segment-specific hindbrain and pharyngeal arch reporter expression**. **A-R**, zebrafish elements from the lamprey (**A-J, M, O, Q**) and jawed vertebrate (**K, L, N, P, R**) CNE sets drive GFP expression in the hindbrain and pharyngeal arches. Elements: Evi1_40224 (**A, B**), Tshz3_43509 (**C, D)**, NR2F2_27254 (**E, F)**, Pax2_217 (**G**, dorsal view: **H**), ZNF503_32799 (**I, J**), Nkx6-1_4281 (**K, L**), Tshz3_24804 (**M**), Pax9_2099 (**N**), TshZ3_24805-6 (**O**), FoxP1_886 (**P**), Tshz3_24807 (**Q**), BCL11A_2554 (**R**). Expression in the hindbrain is often restricted to certain rhombomeres, as shown by comparison with r3r5 RFP expression (**B, D, F, H, J, L**). Tshz3_24807 drives expression in the trunk musculature (**Q**). Elements show temporal variation in reporter expression, expressing most strongly at 24-30hpf (**C, D**), 48-54hpf (**A, B, E, F, I, J, Q**) or 72-78hpf (**G, H, K, L, M, N, O, P, R**). hb: hindbrain; pa: pharyngeal arches; m: muscle.

Next, we tested whether Pbx-Hox motifs within CNEs associate with segment-specific reporter expression in the hindbrain and pharyngeal arches. To do this, we assayed 21 zebrafish CNEs containing conserved Pbx-Hox motifs for reporter expression in zebrafish. All of these CNEs are conserved across gnathostomes, with 11 also identifiable in lamprey (Additional File 2). Elements were chosen to represent a range of different genes from the lamprey and gnathostome CNE sets. 12 of these 21 elements consistently up-regulate patterns of reporter expression, comprised of 8 from the lamprey set and 4 from the gnathostome set. It should be noted that some of the elements from which no consistent expression patterns were obtained may act as enhancers *in-vivo*, but not in our transient transgenic reporter assay, possibly due to being taken out of their genomic context. Remarkably, 11 of the 12 GFP-expressing elements (91.6%) drive expression either in the hindbrain, pharyngeal arches or both, with one element expressing in the trunk musculature (Figure 4). In support of the hypothesis that these elements are directly regulated by specific Hox proteins, which have segmentally-restricted expression patterns, the majority of the elements expressing in the hindbrain do so in particular rhombomeres, as shown by comparison with r3r5 RFP expression (Figure 4). Hindbrain reporter expression driven by these elements is often further restricted dorso-ventrally (e.g. Nkx6-1_4281), medio-laterally (e.g. Pax2_217) and temporally (e.g. Tshz3_43509).

We next examined functional data from the VISTA Enhancer Browser (EB). Compared to shuffled motifs, the KR motif was found to be significantly enriched in those elements annotated as hindbrain positive as well as those positive for either hindbrain, branchial arch or cranial nerve expression (Table 1). Investigating those EB elements that overlap CNEs from the CONDOR set, we found significant enrichment for the KR motif in those with hindbrain expression (HB+, 64 motifs in 112 kb) compared with those with no hindbrain annotation (HB-, 85 in 238 kb) (chi-square p = 0.0042). We then focused upon those sub-regions within EB enhancers that align directly with CNEs. Within these deeply conserved regions, there was more than two-fold enrichment for the stringent Pbx-Hox motif (30 occurrences in 24990 bp of HB+ elements compared with 32 occurrences in 60341 bp of HB- elements; p = 0.001). Importantly, this enrichment demonstrates that Pbx-Hox motifs in ancient CNEs show a correlation with hindbrain reporter expression. We also analysed a smaller dataset from the cneBrowser [21] that contains evolutionarily conserved enhancers associated with genes expressed in forebrain and hindbrain during zebrafish development. Although only 18 of 146 enhancers are annotated as hindbrain positive, 7 out of a total of 17 identified KR motifs reside in hindbrain positive enhancers (p = 3 × 10^-4^) (Table 1).

### CNEs containing Pbx-Hox motifs are associated with genes that have roles in A-P patterning of the hindbrain and head

We have examined the distribution of Pbx-Hox motifs across CNEs of different genes, to ask whether genes with the highest enrichment of Pbx-Hox motifs in their CNEs have roles in hindbrain or pharyngeal arch patterning (Table 2 and Additional file 6). In keeping with the common use of auto-regulation in gene-regulatory networks [45], we find the CNEs of the *HOXD *cluster and the Hox co-factors, *PBX3 *and *MEIS2*, to be amongst those with the highest number of these motifs. Many of the other genes with the highest density of Pbx-Hox motifs in their CNEs have characterised roles in anterior-posterior (A-P) head patterning and show segment specific patterns of expression during development. For instance, the ZNF503/703 (Nlz1 and Nlz2) zinc-finger proteins are essential for specification of rhombomere 4 in zebrafish [46,47]. The orphan nuclear receptor genes *NR2F1/2 *(COUP-TF1/2) are negative transcriptional regulators involved in the retinoic acid signalling pathway, which has a key role in A-P patterning of the hindbrain and pharyngeal arches [48]. The members of the teashirt protein family (*TSHZ1, 2 *and *3*) show segment-specific hindbrain expression [49], *Tshz1 *being essential for segmentally restricted gene expression in the hindbrain and pharyngeal arches of frog and mouse [50,51].

**Table 2:** Frequency of KR motifs in CNEs at different gene loci

GENE	# KR motifs in test set	Length of CNE seq for locus (kb)	#hits per kb	# hits in control set (mean)	standard deviation	z-score	p-value
ZNF503	36	27.781	1.30	3.18	1.76	18.62	0.00E+00
TSHZ3	30	23.323	1.29	3.09	1.77	15.23	0.00E+00
IRX5	27	37.059	0.73	5.39	2.33	9.29	0.00E+00
IRX2	21	23.981	0.88	3.10	1.80	9.95	0.00E+00
TSHZ1	16	10.351	1.55	1.63	1.32	10.93	0.00E+00
PBX3	16	17.886	0.89	1.89	1.35	10.44	0.00E+00
HOXD9	16	17.77	0.90	2.19	1.44	9.59	0.00E+00
NR2F2	16	18.99	0.84	2.52	1.59	8.49	0.00E+00
NR2F1	16	25.655	0.62	3.72	1.84	6.67	2.53E-11
MEIS2	16	24.553	0.65	3.42	1.91	6.59	4.49E-11
ZFHX1B	13	23.275	0.56	3.13	1.72	5.73	9.86E-09
SALL3	12	11.405	1.05	1.43	1.21	8.76	0.00E+00
FOXP1	12	15.857	0.76	1.73	1.24	8.25	2.22E-16
MAF	11	7.334	1.50	1.15	1.10	8.95	0.00E+00
NKX6-1	10	6.853	1.46	0.82	0.92	9.94	0.00E+00

Details are shown for the 15 gene loci from the CONDOR CNE set with the highest number of Pbx-Hox KR motifs in their CNEs, showing enrichment relative to shuffled CNE sets (Methods and Additional File 6). For each gene locus, the number of Pbx-Hox KR motifs in the associated CNEs is given. The number of Pbx-Hox KR motifs per kb of CNE sequence for each locus (column 4) is calculated by dividing the number of Pbx-Hox KR motifs in the CNEs of that locus (column 2) by the total combined length of the CNEs in that locus (column 2). Control sets were generated by zero order Markov shuffling of CNEs at each locus in 1000 randomisations (Methods). Some gene loci also contain other genes besides the one after which they are named, for instance the IRX5 locus contains Irx3, Irx5 and Irx6.

There is good agreement between the genes highlighted by our *in-silico *binding-site search and by microarray screens for downstream targets of *hoxb1 *in rhombomere 4 of zebrafish [52] and mouse [53]. Specifically, the expression levels of *znf503, tshz2, evi1, zic4, shox*, and *meis2.1 *are decreased upon knock-down of *HoxB1 *in zebrafish, with *Znf503, Nkx6-1, Atbf1 *and *Mab21l2 *down-regulated in *HoxB1*-/- mouse embryos. Accordingly, the CNEs around each of these genes are enriched in Pbx-Hox motifs (Table 2 and Additional File 6). Thus, both microarray datasets are consistent with our prediction that Pbx-Hox motifs in CNEs represent direct regulatory links between Hox genes and their targets during development.

## Discussion

### Discovery of Pbx-Hox motif enrichment is a further step toward de-coding CNEs

Despite a pervasive assumption that CNEs bind transcription factors in order to elicit gene activation, there is, perhaps surprisingly, very little direct evidence to confirm this. We sought to identify TFBS motifs in CNEs through phylogenetic footprinting, reasoning that the relatively high divergence of lamprey CNEs would highlight important motifs. The utility of this approach is confirmed by our identification of conserved Pbx-Hox and Meis TFBS motifs in CNEs. The enrichment of the Pbx-Hox TFBS motif in the jawed vertebrate CNE set reveals this motif to be a regulatory signature that is utilised by a large proportion of highly conserved *cis*-regulatory elements (the 6, 693 CONDOR CNEs contain 562 KR motifs and 1416 TGATNNAT motifs). Whilst enriched motifs identified in mammalian conserved elements include a few that show partial overlap with variants of the Pbx-Hox consensus motif [23,24], the link between those enriched motifs and Hox factors had not been made, and their strong enrichment in more ancient CNEs had not been characterised. This enrichment agrees with the crucial, conserved roles of Hox factors in development of the vertebrate body plan. Indeed, the association of these motifs with hindbrain and pharyngeal arch enhancer function is in keeping with the characterised roles of Pbx, Hox and Meis factors in patterning these domains.

Despite the crucial roles of Hox factors in patterning the vertebrate embryo, relatively few downstream target genes, other than the *hox *genes themselves, have been identified. Our data suggests that Pbx-Hox motifs in CNEs can identify such targets. The striking manner in which Pbx-Hox and Meis TFBS motifs are highlighted as conserved sequence blocks in multiple alignments, especially when lamprey sequences are included, leads us to predict that this footprinting approach will be useful for further deciphering the regulatory code within vertebrate enhancers. In combination, our *in-silico *and functional analyses form an important link between well characterised *cis*-regulatory motifs and a large proportion of relatively uncharacterised ancient CNEs, helping to better place these elements within a developmental context. This represents a significant further step in systematically de-coding the enhancers responsible for development of the vertebrate body plan and highlights the utility of the lamprey as a model system for investigating vertebrate gene regulation.

The diversity of expression patterns driven by our tested elements suggests that Pbx-Hox TFBSs are just one component of a complex *cis*-regulatory logic encoded within these enhancers. Whilst responding to A-P patterning cues by interacting with particular Hox factors through Pbx-Hox TFBSs, these elements concomitantly determine the tissues in which they are active (e.g. hindbrain vs pharyngeal arch) and limit the expression patterns dorso-ventrally, medio-laterally and temporally. An example of this is the CNE Pax2_403, which drives reporter expression that is restricted to a ventro-lateral population of neurons in r2-3 of the hindbrain (Figure 4). Furthermore, whilst some of our functionally characterised CNEs drive reporter expression in domains with sharp boundaries that are co-incident with rhombomere boundaries -similar to that of previously characterised Pbx-Hox regulated elements - this is not the case for all of them. This could be due to the Pbx-Hox input establishing a competence for the enhancer to drive expression within particular rhombomeres, which is further restricted to specific sub-domains within the rhombomeres by the influence of other regulatory inputs to the enhancer. This would result in expression domains that do not encompass the whole area of expression of the regulating Hox factor. It is likely that the reason why many previously characterised Pbx-Hox regulated elements show expression domains across whole rhombomeres and with tight boundaries co-incident with rhombomere boundaries is that the majority of these elements are regulating Hox factors, and thus setting up or maintaining the rhombomere-specific Hox expression patterns. Many of the elements described in this study may be acting downstream of this Hox network, utilising these AP patterning cues along with other cues to further pattern the hindbrain. The tissue specificity of these enhancers, as well as the restriction of expression to specific domains and time points, is presumably due to other factors acting as specifiers by binding to nearby TFBSs. Identifying these specifiers and characterising their TFBSs, as well as the nature of their interactions with Hox factors, are key tasks toward understanding the *cis*-regulatory logic underlying vertebrate development. The set of putative Hox-responsive *cis*-regulatory elements identified in this study provides a powerful resource that will facilitate efforts toward this end.

Our expression data from the mutated versions of zebrafish CNE 3299 suggests that the multiple Pbx-Hox and Meis sites predicted in this enhancer may interact with each other, to co-operatively modulate and restrict reporter expression. The two clusters of Pbx-Hox and Meis motifs do not contribute equally to the expression driven by this enhancer in the hindbrain and pharyngeal arch neural crest. The second Pbx-Hox and Meis motif cluster appears to be necessary for the general function of this enhancer, as its mutation results in the loss of reporter expression in both hindbrain and neural crest. In contrast, the first Pbx-Hox and Meis motif cluster appears to be necessary (but not sufficient) for neural crest expression, but not for hindbrain expression. Conversely, it appears to restrict the hindbrain expression, as reporter expression is seen more anteriorly when this cluster is mutated. This is reminiscent of interactions between Pbx-Hox and Meis/Prep binding sites within a Hoxb1 enhancer, which direct expression of this gene to r4 the hindbrain in mouse and chick [54]. In that case, it was found that the formation of a Pbx-Hox-Meis/Prep ternary complex on Pbx-Hox and Meis sites within this enhancer could be restricted by the binding of a Pbx1-Prep1 heterodimer to a nearby site, thus limiting the expression driven by this enhancer to r4. This highlights the complexity of the regulatory interactions between transcription factors that are likely to bind to CNEs, a complexity that could well underlie their high sequence constraint.

### A potential role for CNEs in the evolution of vertebrate head patterning

A strength of identifying conserved *cis*-regulatory elements is that they can provide compelling evidence for conserved GRNs. Our reporter assay data from zebrafish and lamprey embryos clearly demonstrate functional conservation of enhancers shared between the most distantly related extant vertebrate lineages. We deduce that all vertebrates share aspects of a GRN for hindbrain patterning, downstream of nested Hox expression. As the sea lamprey is from a vertebrate lineage that diverged prior to the evolution of many jawed vertebrate innovations, such as paired appendages and jaws [55], we predict that the lamprey reporter assay will be a crucial tool for investigating the gene regulatory changes involved in vertebrate evolution.

Without detailed knowledge of the function or mechanism of action of CNEs it has been difficult to derive scenarios of how they evolved and became fixed in vertebrate genomes. The findings from our *in silico *and functional analyses, coupled with previous characterisation of Pbx-Hox and Meis transcription-factor complexes, enable us to propose a hypothesis regarding the role of a large number of CNEs in vertebrate evolution. Recognising the same TFBS motifs in worms, flies and vertebrates, the Pbx, Hox and Meis factors are part of an ancient regulatory language shared across bilaterians [17,36,37,56,57]. Nevertheless, none of the CNEs containing these motifs are identifiable in invertebrate genomes, leading us to speculate that many of these elements may have arisen in the vertebrate lineage. Accordingly, our functional data suggest that many of these CNEs have roles in patterning an elaborate head and brain - key vertebrate innovations [54]. We hypothesise that the fundamental role of head patterning in vertebrates led to the functional conservation of these elements and that their reliance upon the precise organisation of TFBSs necessitated their strict sequence conservation. The mechanisms through which new *cis*-regulatory elements arise in the genome are still largely unresolved. In this case, the finding that simple Pbx-Hox sites are sufficient to drive robust and specific, but modifiable, expression [17] hints that these particular TFBSs may pioneer new *cis*-regulatory elements, functioning as one of the fundamental seeds from which many CNEs were able to grow.

## Conclusions

The finding that vertebrate CNEs are highly enriched for Pbx-Hox binding-site motifs represents a further step toward de-coding ancient vertebrate enhancers. Coupled with our experimental data, this enables a large proportion of these elements to be more firmly placed into a developmental context and reveals ancient gene regulatory network interactions for hindbrain and head patterning that were present in ancestral vertebrates. Finally, our findings lead us to hypothesise that the evolution of many of these CNEs contributed to the elaboration of the vertebrate hindbrain and the branchial region of the head.

## Methods

### Identification of CNEs

6, 693 non-redundant human CNEs (average length 116 bp) were retrieved from the CONDOR database [43] at http://condor.nimr.mrc.ac.uk (Additional File 7). We used these to search lamprey sequence reads available from the NCBI trace server at http://www.ncbi.nlm.nih.gov/Traces/trace.cgi with sensitive parameters (-W 7 -q -1 -e 5e-4) as described previously [28]. The lamprey trace sequences were searched because they represent a greater coverage of the lamprey genome than the publicly available draft genome assembly, which consists of many short contigs and thus provides little advantage with regard to identification of conserved syntenic regions. Lamprey sequences satisfying the initial parametric threshold were further analysed for contamination, and those with > 90% homology to human or chicken across the whole read (i.e. extending outside the evolutionarily conserved region in other vertebrates) were removed.

### Alignments

The sequences of human, fugu and most zebrafish CNEs were retrieved from the CONDOR database [57]. Additional zebrafish CNEs that were not previously included in the CONDOR database due to absence from earlier assemblies were identified using BLAST against a more recent zebrafish genome assembly (Zv8 release 58). Sequences in each alignment were clipped to the same size to prevent unaligned edges. To align the sequences we used ClustalW version 1.83. These alignments formed the lamprey CNE set (comprised of alignments of CNEs from human, fugu, zebrafish and lamprey) and the gnathostome CNE set (containing alignments of CNEs from human, fugu and zebrafish). As a control, for each CNE we also generated 1000 multiple alignments by randomly shuffling the columns of each alignment using the seqboot implementation in Phylip version 3.67. The sequences of lamprey and zebrafish CNEs in these datasets are given in Additional File 8 and Additional File 9 respectively. The sequences of CNEs from the EB and cneBrowser datasets are given in Additional File 10 and Additional File 11 respectively.

### Scanning CNEs and control sets for Pbx-Hox motifs

We searched for Pbx-Hox motifs in two different types of datasets. Firstly, in multiple sequence alignments of CNEs from the CONDOR CNE database (the lamprey and gnathostome CNE sets). Secondly, in datasets consisting of sequences from just one species (the EB CNEs, shark CNEs, human elements from the CONDOR CNE set, cneBrowser CNEs). The two different types of datasets required different types of control to test for Pbx-Hox motif enrichment. For the alignment sets, we generated control sets of shuffled alignments. To find evolutionarily conserved Pbx-hox motifs (TGATNNAT and TGATNNATKR) we employed the software Cis-Finder [42] on our two alignment sets and their respective shuffled alignment controls. A motif match was only considered if it matched all aligned species and occurred at the exact same aligned position. For the single species sequence sets we generated shuffled motifs, based upon the KR motif, as a control to search across the same sets. In parallel we also employed a *de-novo *motif finding strategy implemented in Cis-Finder on the CONDOR CNE set. It scans a set of DNA sequences for over-represented position frequency matrices (PFMs), clusters these and then estimates significance using the false discovery rate [42]. The TGATNNAT and TGATNNATKR motif occurances in the CONDOR CNE set are detailed in Additional File 12 and Additional File 13.

To characterise the frequency of Pbx-Hox KR motifs in different gene loci, we used the CONDOR CNE set, in which CNEs are grouped according to gene locus as specified in the CONDOR database [57]. For each gene locus, we counted the frequency of Pbx-Hox KR motifs in the associated CNEs and compared this to the average frequency in 1000 sets of randomised versions of CNEs from that locus. A markov chain model of order zero was used to generate shuffled sequences. To model DNA sequences, 4 states (A, C, G, T) and 4 transitions were used. Transition probabilities were retrieved from the CNE set by calculating the relative frequencies of the bases.

### Measuring relative enrichment of Pbx-Hox motifs

To measure the enrichment, we compared the occurrence of Pbx-Hox motifs in a test set against shuffled versions (Table 1), calculated mean and standard deviation and generated z-scores. The z-scores were then transformed into p-values under a normal distribution model. We also counted Pbx-Hox occurrence and shuffled versions in a number of control regions and across the whole human genome (Additional File 4).

### Overlap with other evolutionarily conserved 'enhancer' sets

There is inevitably some overlap between the different sets of evolutionarily conserved sequences. 482/1307 EB human sequences overlap 994 CONDOR CNEs, covering a total of 146226 bases (7.4% of the EB sequence; 18.8% of CNE sequence). 1632 human sequences identified through comparison with *Callorhinchus milii *[13] overlap 2172 CONDOR CNEs, covering a total of 271260 bases (26.5% of the *Callorhinchus *dataset; 34.9% of CNEs). Finally, 69/146 zebrafish cneBrowser sequences overlap 83 CONDOR CNEs, covering a total of 11496 bases (20.5% of the cneBrowser sequence; 1.5% of CNE sequence).

### Zebrafish transgenesis

CNEs were amplified from zebrafish and lamprey genomic DNA by PCR, sub-cloned into the Pcr8/GW/TOPO vector (Invitrogen) and then into a Tol2 construct (pGW_cfosEGFP) [29,58,59], using the Gateway LR Clonase II enzyme (Invitrogen). The Tol2 reporter assay was performed as described previously [29]. Transient transgenic zebrafish embryos were screened for GFP expression at 24-30hpf, 48-54hpf and 72-78hpf using a Leica M165FC microscope and photographs taken with a Leica DFC310FX camera. Expression patterns were deemed consistent when found in > 20% of founders, consistent with previous studies [25,60].

### CNE Mutagenesis

Mutations in zebrafish CNE 3299 were introduced by PCR from genomic DNA with primers containing the desired mutations either through conventional PCR (for sub1) or megaprimer PCR (for sub2) [61]. Mutated CNE PCR products were then cloned for zebrafish transgenesis as described above.

### Lamprey transgenesis

The transgenesis protocol was based upon that developed in Xenopus [62]. Lamprey CNEs were amplified from genomic DNA and cloned into the cFos-I-sceI-EGFP plasmid, which contains the mouse cFos minimal promoter and EGFP coding sequence flanked by I-sceI restriction sites. Plasmids were extracted using the EndoFree Plasmid Maxi Kit (Qiagen) and eluted with water through QIAQuick columns (Qiagen). Fresh restriction digests (20 μl containing 400 ng plasmid, 15 units I-SceI enzyme (NEB), 1 × I-SceI buffer + BSA, digested for 40 minutes at 37°C) were micro-injected into 5-6hpf lamprey embryos using a Pico-Spritzer with drop volume of 2-3 nl. Lamprey husbandry was performed as described previously [63]. Embryos were screened for GFP expression between embryonic days 7-16. Typical survival rates ranged from 20-50% of injected embryos. The promoter alone drives highly mosaic background expression in the ectoderm in roughly 50% of surviving embryos. Enhancer-specific expression was seen in approximately 10% of surviving embryos.

## Abbreviations

CNE: conserved non-coding element; TFBS: transcription factor binding site; GRN: gene regulatory network; EB: enhancer browser (dataset); A-P: anterior-posterior.

## Authors' contributions

Conceived and designed the experiments: HJP and GE. Performed the experiments: HJP. Conceived and designed bio-informatic analyses: PP, HJP and GE. Performed bio-informatic analyses: PP and GE. Analysed the data: HJP, PP and GE. Supplied materials, reagents and lamprey expertise: MB and TS-S. Wrote the paper: HJP, PP and GE. All authors read and approved the final manuscript.

## Supplementary Material

Additional file 1 **Genomic sequences used to generate MLAGAN alignment**. The genomic sequences of human, fugu, zebrafish and lamprey used to generate the MLAGAN alignment in this study. Click here for file

Additional file 2 **CNEs functionally tested for this study**. The sequences of the CNEs of zebrafish (dr) and lamprey (pm) that were tested by reporter assay in zebrafish embryos for this study. Click here for file

Additional file 3 **The frequency of Pbx-Hox motifs in different test sets**. A table describing the frequency of Pbx-Hox motifs in different test sets, compared with 1000 randomised controls. Click here for file

Additional file 4 **The frequency of KR motifs in different control sets**. A table listing the frequency of KR motifs, compared to shuffled versions, in different control sets. Click here for file

Additional file 5 **The frequency of Meis motifs in Human CNEs**. A table listing the frequency of Meis motifs, compared to shuffled versions in human CONDOR CNEs. Click here for file

Additional file 6 **The frequency of KR motifs in CNEs at different gene loci**. A table listing the frequency of KR motifs in CNEs at different gene loci. Click here for file

Additional file 7 **The CONDOR CNE set**. 6693 non-redundant human CNE sequences, shared between human and fugu, retrieved from the CONDOR database. Click here for file

Additional file 8 **Lamprey CNEs**. Lamprey sequences of 246 CNEs shared between jawed vertebrates and lamprey (from the lamprey CNE alignment set). Click here for file

Additional file 9 **Zebrafish CNEs**. Zebrafish sequences of 4259 CNEs shared between human, fugu and zebrafish (from the gnathostome CNE alignment set). Click here for file

Additional file 10 **CNEs from the VISTA Enhancer Browser**. 1307 human sequences from the VISTA Enhancer Browser http://enhancer.lbl.gov/. Click here for file

Additional file 11 **CNEs from the cneBrowser**. 146 functionally tested zebrafish sequences from the cneBrowser http://bioinformatics.bc.edu/chuanglab/cneBrowser/#home. Click here for file

Additional file 12 **TGATNNAT motif hits on the human CONDOR CNEs**. Hits of the TGATNNAT motif on the human CONDOR CNEs listed by CNE with details of the start and finish of the motif within the CNE. Click here for file

Additional file 13 **TGATNNATKR motif hits on the human CONDOR CNEs**. Hits of the TGATNNATKR motif on the human CONDOR CNEs listed by CNE with details of the start and finish of the motif within the CNE. Click here for file
