# Supplementary material for: Ancient Pbx-Hox signatures define hundreds of vertebrate developmental enhancers
Source: BMC Genomics. 2011 Dec 30;12:637. doi: 10.1186/1471-2164-12-637 (PMC3261376; doi:10.1186/1471-2164-12-637)
Supplement: Additional file 3 — The frequency of Pbx-Hox motifs in different test sets. A table describing the frequency of Pbx-Hox motifs in different test sets, compared with 1000 randomised controls. [file 1471-2164-12-637-S3.DOC]

**Frequency of Pbx-Hox motifs in different test sets, compared with 1000 randomised controls (see Methods).**

lamp_bs_NN – TGATNNAT motifs in lamprey:human:fugu:zebrafish CNE alignments

zf_bs_NN – TGATNNAT motifs in human:fugu:zebrafish CNE alignments

lamp_bs_KR – TGATNNATKR motifs in lamprey:human:fugu:zebrafish CNE alignments

zf_bs_KR – TGATNNATKR motifs in human:fugu:zebrafish CNE alignments

hs_mc0_NN – TGATNNAT motifs in human CONDOR CNEs with zero order markov shuffling

hs_mc0_KR – TGATNNATKR motifs in human CONDOR CNEs with zero order markov shuffling

| *test vs control* | *Number of CNEs in test set* | *Number of motif hits in test set* | *CNEs with at least one hit in test set* | *Number of Motif hits in controls*  *(mean)* | *CNEs with at least one hit in controls (mean)* | *standard deviation* | *z-score* | *p-value assuming normal distribution* |
| --- | --- | --- | --- | --- | --- | --- | --- | --- |
| lamp_bs_NN | 246 | 61 | 47 | 2.73 | 2.65 | 1.70 | 34.29 | 0 |
| zf_bs_NN | 4259 | 712 | 591 | 78.73 | 75.23 | 8.63 | 73.42 | 0 |
|  |  |  |  |  |  |  |  |  |
| lamp_bs_KR | 246 | 21 | 19 | 0.43 | 0.43 | 0.67 | 30.75 | 0 |
| zf_bs_KR | 4259 | 269 | 250 | 15.11 | 15.02 | 3.90 | 65.16 | 0 |
|  |  |  |  |  |  |  |  |  |
| hs_mc0_NN | 6693 | 1416 | 1158 | 416.81 | 390.25 | 21.37 | 46.75 | 0 |
| hs_mc0_KR | 6693 | 562 | 522 | 102.82 | 101.82 | 9.91 | 46.35 | 0 |
