# Supplementary material for: Ancient Pbx-Hox signatures define hundreds of vertebrate developmental enhancers
Source: BMC Genomics. 2011 Dec 30;12:637. doi: 10.1186/1471-2164-12-637 (PMC3261376; doi:10.1186/1471-2164-12-637)
Supplement: Additional file 4 — The frequency of KR motifs in different control sets. A table listing the frequency of KR motifs, compared to shuffled versions, in different control sets. [file 1471-2164-12-637-S4.DOC]

**Frequency of KR motifs, compared to shuffled versions, in different control sets.** Human chromosomal regions were randomly chosen.

|  |  |  | |  | |  | |  | |  | |  |
| --- | --- | --- | --- | --- | --- | --- | --- | --- | --- | --- | --- | --- |
| **motif** | **humchr6 control** | | **humchr12 control** | | **humchr1 15-19Mb control** | | **250kb hoxd region** | | **Human genome** | |  | |
| **TGATNNATKR** | **151** | | **122** | | **441** | | **45** | | **530509** | |  | |
| TGTANNATKR | 152 | | 143 | | 593 | | 35 | | 543258 | |  | |
| GTATNNATKR | 126 | | 109 | | 290 | | 29 | | 442772 | |  | |
| GTTANNATKR | 131 | | 115 | | 376 | | 37 | | 475051 | |  | |
| TTGANNATKR | 180 | | 149 | | 417 | | 41 | | 626755 | |  | |
| TTAGNNATKR | 176 | | 168 | | 413 | | 31 | | 545811 | |  | |
| ATGTNNATKR | 175 | | 165 | | 523 | | 43 | | 765597 | |  | |
| ATTGNNATKR | 143 | | 136 | | 415 | | 52 | | 550250 | |  | |
| AGTTNNATKR | 171 | | 133 | | 445 | | 40 | | 547730 | |  | |
| TAGTNNATKR | 109 | | 112 | | 205 | | 25 | | 364553 | |  | |
| TATGNNATKR | 123 | | 130 | | 332 | | 33 | | 522892 | |  | |
| GATTNNATKR | 146 | | 144 | | 459 | | 31 | | 521158 | |  | |
| TGATNNTAKR | 109 | | 121 | | 292 | | 32 | | 489556 | |  | |
| TGTANNTAKR | 236 | | 221 | | 432 | | 48 | | 790068 | |  | |
| GTATNNTAKR | 108 | | 91 | | 209 | | 25 | | 372793 | |  | |
| GTTANNTAKR | 125 | | 115 | | 234 | | 31 | | 419041 | |  | |
| TTGANNTAKR | 154 | | 148 | | 351 | | 40 | | 548745 | |  | |
| TTAGNNTAKR | 102 | | 119 | | 217 | | 22 | | 371159 | |  | |
| ATGTNNTAKR | 196 | | 171 | | 379 | | 36 | | 615961 | |  | |
| ATTGNNTAKR | 129 | | 121 | | 254 | | 33 | | 474064 | |  | |
| AGTTNNTAKR | 163 | | 148 | | 309 | | 34 | | 475571 | |  | |
| TAGTNNTAKR | 127 | | 84 | | 222 | | 15 | | 409042 | |  | |
| TATGNNTAKR | 179 | | 197 | | 403 | | 48 | | 694915 | |  | |
| GATTNNTAKR | 110 | | 103 | | 330 | | 26 | | 413929 | |  | |
|  |  | |  | |  | |  | |  | |  | |
| **mean** | 146.71 | | 136.04 | | 355.88 | | 34.67 | | 521299 | |  | |
| **S.D** | 33.15 | | 32.03 | | 103.86 | | 8.92 | | 112360 | |  | |
| **z-score for pbxhox** | 0.13 | | 0.43 | | 0.817 | | 1.16 | | 0.082 | |  | |
| **p-value** | N/S | | N/S | | N/S | | N/S | | N/S | |  | |
