# Supplementary material for: Ancient Pbx-Hox signatures define hundreds of vertebrate developmental enhancers
Source: BMC Genomics. 2011 Dec 30;12:637. doi: 10.1186/1471-2164-12-637 (PMC3261376; doi:10.1186/1471-2164-12-637)
Supplement: Additional file 5 — The frequency of Meis motifs in Human CNEs. A table listing the frequency of Meis motifs, compared to shuffled versions in human CONDOR CNEs. [file 1471-2164-12-637-S5.DOC]

**Frequency of Meis motifs, compared to shuffled versions in 6693 Human CNEs.**

| *Shuffled MEIS motifs* | *# CNEs with at least one MEIS hit* | *Total # MEIS hits* |
| --- | --- | --- |
| **TGACAR** | **1825** | **2361** |
| ATGCAR | 1300 | 1617 |
| CAGATR | 1220 | 1412 |
| TGCAAR | 1207 | 1573 |
| CTGAAR | 1193 | 1393 |
| TCAGAR | 1151 | 1348 |
| AAGCTR | 1007 | 1166 |
| ATCAGR | 976 | 1124 |
| AATGCR | 962 | 1151 |
| ACAGTR | 958 | 1082 |
| ATGACR | 958 | 1095 |
| GTCAAR | 920 | 1044 |
| ACTGAR | 914 | 1020 |
| TAGCAR | 888 | 1003 |
| AGCATR | 865 | 1011 |
| AACTGR | 861 | 952 |
| CAATGR | 854 | 975 |
| CAGTAR | 853 | 944 |
| CATGAR | 829 | 997 |
| GCAATR | 806 | 908 |
| ACATGR | 797 | 879 |
| AGTCAR | 781 | 874 |
| TCAAGR | 772 | 852 |
| CAAGTR | 729 | 798 |
| TACAGR | 714 | 803 |
| GCATAR | 688 | 784 |
| GATCAR | 682 | 824 |
| GACATR | 668 | 730 |
| GCTAAR | 660 | 731 |
| AGCTAR | 657 | 776 |
| TGAACR | 620 | 680 |
| AGACTR | 582 | 627 |
| GAACTR | 582 | 628 |
| AAGTCR | 548 | 589 |
| TAAGCR | 546 | 593 |
| AGATCR | 513 | 553 |
| GTACAR | 509 | 629 |
| GAATCR | 494 | 527 |
| ATAGCR | 489 | 531 |
| CTAAGR | 403 | 424 |
| GACTAR | 397 | 421 |
| CATAGR | 387 | 403 |
| GTAACR | 386 | 416 |
| CTAGAR | 381 | 431 |
| TAGACR | 381 | 411 |
| ACTAGR | 370 | 391 |
| AGTACR | 339 | 359 |
| GATACR | 329 | 343 |
| AATCGR | 221 | 230 |
| AACGTR | 173 | 178 |
| TAACGR | 171 | 178 |
| CGAATR | 164 | 169 |
| CGATAR | 156 | 161 |
| ATCGAR | 148 | 152 |
| TCGAAR | 141 | 152 |
| CGTAAR | 138 | 141 |
| ACGATR | 132 | 135 |
| ATACGR | 119 | 121 |
| ACGTAR | 112 | 117 |
| TACGAR | 100 | 103 |
| mean | 628.77 | 717 |
| S.D. | 364.01 | 452.28 |
| z-score for meis motif **TGACAR** | 3.28 | 3.63 |
| p-value | 0.0005 | 0.0001 |
