# Supplementary material for: Ancient Pbx-Hox signatures define hundreds of vertebrate developmental enhancers
Source: BMC Genomics. 2011 Dec 30;12:637. doi: 10.1186/1471-2164-12-637 (PMC3261376; doi:10.1186/1471-2164-12-637)
Supplement: Additional file 6 — The frequency of KR motifs in CNEs at different gene loci. A table listing the frequency of KR motifs in CNEs at different gene loci. [file 1471-2164-12-637-S6.DOC]

**Frequency of KR motifs in CNEs at different gene loci.** Control sets are generated by zero order Markov shuffling of CNEs at each locus in 1000 randomisations (Methods). Some gene loci include other genes besides the one after which the locus is named.

| GENE | Number of KR motifs in test set | Length of CNE seq for locus (kb) | #hits per kb | # hits in control set (mean) | standard deviation | z-score | p-value |
| --- | --- | --- | --- | --- | --- | --- | --- |
| ZNF503 | 36 | 27.781 | 1.30 | 3.18 | 1.76 | 18.62 | 0.00E+00 |
| TSHZ3 | 30 | 23.323 | 1.29 | 3.09 | 1.77 | 15.23 | 0.00E+00 |
| IRX5 | 27 | 37.059 | 0.73 | 5.39 | 2.33 | 9.29 | 0.00E+00 |
| IRX2 | 21 | 23.981 | 0.88 | 3.10 | 1.80 | 9.95 | 0.00E+00 |
| TSHZ1 | 16 | 10.351 | 1.55 | 1.63 | 1.32 | 10.93 | 0.00E+00 |
| PBX3 | 16 | 17.886 | 0.89 | 1.89 | 1.35 | 10.44 | 0.00E+00 |
| HOXD9 | 16 | 17.77 | 0.90 | 2.19 | 1.44 | 9.59 | 0.00E+00 |
| NR2F2 | 16 | 18.99 | 0.84 | 2.52 | 1.59 | 8.49 | 0.00E+00 |
| NR2F1 | 16 | 25.655 | 0.62 | 3.72 | 1.84 | 6.67 | 2.53E-11 |
| MEIS2 | 16 | 24.553 | 0.65 | 3.42 | 1.91 | 6.59 | 4.49E-11 |
| ZFHX1B | 13 | 23.275 | 0.56 | 3.13 | 1.72 | 5.73 | 9.86E-09 |
| SALL3 | 12 | 11.405 | 1.05 | 1.43 | 1.21 | 8.76 | 0.00E+00 |
| FOXP1 | 12 | 15.857 | 0.76 | 1.73 | 1.24 | 8.25 | 2.22E-16 |
| MAF | 11 | 7.334 | 1.50 | 1.15 | 1.10 | 8.95 | 0.00E+00 |
| NKX6-1 | 10 | 6.853 | 1.46 | 0.82 | 0.92 | 9.94 | 0.00E+00 |
| BCL11A | 10 | 13.643 | 0.73 | 2.14 | 1.43 | 5.48 | 4.15E-08 |
| FOXP2 | 10 | 17.844 | 0.56 | 2.30 | 1.51 | 5.09 | 3.49E-07 |
| EBF3 | 10 | 26.18 | 0.38 | 3.22 | 1.79 | 3.78 | 1.55E-04 |
| MEIS1 | 9 | 9.298 | 0.97 | 1.46 | 1.21 | 6.21 | 5.21E-10 |
| NR4A2 | 9 | 14.765 | 0.61 | 1.51 | 1.24 | 6.03 | 1.60E-09 |
| ZNF703 | 8 | 2.991 | 2.67 | 0.37 | 0.60 | 12.68 | 0.00E+00 |
| GLI3 | 8 | 3.432 | 2.33 | 0.50 | 0.70 | 10.67 | 0.00E+00 |
| SHOX2 | 8 | 7.615 | 1.05 | 0.97 | 1.00 | 7.05 | 1.85E-12 |
| OTP | 8 | 8.986 | 0.89 | 1.28 | 1.13 | 5.93 | 3.09E-09 |
| DACH1 | 8 | 12.338 | 0.65 | 1.43 | 1.23 | 5.36 | 8.28E-08 |
| ESRRG | 8 | 8.743 | 0.92 | 1.73 | 1.36 | 4.60 | 4.21E-06 |
| ESRRB | 7 | 4.537 | 1.54 | 0.66 | 0.82 | 7.72 | 1.13E-14 |
| EMX2 | 7 | 8.736 | 0.80 | 0.81 | 0.90 | 6.90 | 5.17E-12 |
| TSHZ2 | 7 | 7.221 | 0.97 | 0.86 | 0.93 | 6.58 | 4.85E-11 |
| TCF7L2 | 7 | 11.045 | 0.63 | 1.51 | 1.21 | 4.52 | 6.31E-06 |
| SOX14 | 6 | 4.286 | 1.40 | 0.42 | 0.64 | 8.75 | 0.00E+00 |
| POU3F2 | 6 | 3.297 | 1.82 | 0.47 | 0.69 | 8.02 | 1.11E-15 |
| EVI1 | 6 | 4.015 | 1.49 | 0.65 | 0.82 | 6.55 | 5.84E-11 |
| MAB21L2 | 6 | 5.733 | 1.05 | 0.89 | 0.93 | 5.48 | 4.37E-08 |
| FOG2 | 6 | 11.306 | 0.53 | 1.07 | 1.07 | 4.61 | 4.08E-06 |
| ZIC1 | 6 | 8.332 | 0.72 | 1.14 | 1.07 | 4.54 | 5.57E-06 |
| ATBF1 | 6 | 7.514 | 0.80 | 1.18 | 1.12 | 4.31 | 1.67E-05 |
| SATB1 | 5 | 5.558 | 0.90 | 0.54 | 0.76 | 5.91 | 3.52E-09 |
| HMX2 | 5 | 7.662 | 0.65 | 0.98 | 0.96 | 4.19 | 2.75E-05 |
| PAX2 | 5 | 9.193 | 0.54 | 1.08 | 1.05 | 3.75 | 1.78E-04 |
| TFAP2A | 5 | 11.087 | 0.45 | 1.25 | 1.11 | 3.39 | 7.10E-04 |
| SOX6 | 5 | 10.525 | 0.48 | 1.38 | 1.18 | 3.08 | 2.06E-03 |
| BARHL2 | 5 | 12.98 | 0.39 | 1.41 | 1.17 | 3.08 | 2.07E-03 |
| POU6F2 | 4 | 3.103 | 1.29 | 0.45 | 0.70 | 5.09 | 3.56E-07 |
| PAX9 | 4 | 3.217 | 1.24 | 0.51 | 0.74 | 4.70 | 2.63E-06 |
| UNC4 | 4 | 6.23 | 0.64 | 0.65 | 0.84 | 3.98 | 6.98E-05 |
| PRDM16 | 4 | 3.736 | 1.07 | 0.74 | 0.83 | 3.91 | 9.16E-05 |
| PAX6 | 4 | 4.913 | 0.81 | 0.73 | 0.89 | 3.69 | 2.28E-04 |
| EBF1 | 4 | 4.032 | 0.99 | 0.93 | 0.96 | 3.22 | 1.29E-03 |
| PAX1 | 4 | 9.457 | 0.42 | 1.05 | 1.00 | 2.96 | 3.12E-03 |
| FIGN | 4 | 8.809 | 0.45 | 1.02 | 1.07 | 2.78 | 5.49E-03 |
| FOXB1 | 4 | 8.603 | 0.46 | 1.18 | 1.11 | 2.55 | 1.08E-02 |
| BNC2 | 4 | 10.59 | 0.38 | 1.65 | 1.33 | 1.77 | N/S |
| ZFHX4 | 4 | 12.676 | 0.32 | 1.95 | 1.38 | 1.48 | N/S |
| POU3F1 | 3 | 4.128 | 0.73 | 0.72 | 0.85 | 2.69 | 7.21E-03 |
| POU4F2 | 3 | 5.809 | 0.52 | 0.72 | 0.87 | 2.63 | 8.58E-03 |
| MAB21L1 | 3 | 4.838 | 0.62 | 0.74 | 0.87 | 2.60 | 9.25E-03 |
| ZIC2 | 3 | 7.68 | 0.39 | 0.92 | 0.97 | 2.14 | 3.26E-02 |
| FOXD3 | 3 | 9.839 | 0.30 | 1.29 | 1.13 | 1.52 | N/S |
| PAX5 | 2 | 1.05 | 1.90 | 0.27 | 0.51 | 3.42 | 6.26E-04 |
| EN1 | 2 | 2.244 | 0.89 | 0.40 | 0.62 | 2.56 | 1.04E-02 |
| GBX2 | 2 | 2.623 | 0.76 | 0.46 | 0.66 | 2.32 | 2.06E-02 |
| SOX21 | 2 | 5.466 | 0.37 | 0.49 | 0.68 | 2.21 | 2.71E-02 |
| SHOX | 2 | 4.128 | 0.48 | 0.49 | 0.71 | 2.14 | 3.26E-02 |
| SP8 | 2 | 4.61 | 0.43 | 0.51 | 0.71 | 2.12 | 3.44E-02 |
| PHOX2B | 2 | 4.45 | 0.45 | 0.61 | 0.79 | 1.77 | N/S |
| LMO4 | 2 | 6.74 | 0.30 | 0.66 | 0.83 | 1.61 | N/S |
| DLX1 | 2 | 6.28 | 0.32 | 0.70 | 0.81 | 1.60 | N/S |
| SOX5 | 2 | 3.691 | 0.54 | 0.73 | 0.84 | 1.52 | N/S |
| LHX1 | 2 | 4.442 | 0.45 | 0.75 | 0.85 | 1.46 | N/S |
| EYA1 | 2 | 5.536 | 0.36 | 0.81 | 0.89 | 1.34 | N/S |
| BHLHB5 | 2 | 6.937 | 0.29 | 0.96 | 0.97 | 1.07 | N/S |
| CST | 2 | 8.39 | 0.24 | 0.96 | 0.98 | 1.06 | N/S |
| AUTS2 | 2 | 7.036 | 0.28 | 1.01 | 1.02 | 0.97 | N/S |
| SOX11 | 1 | 1.678 | 0.60 | 0.19 | 0.42 | 1.93 | N/S |
| SOX3 | 1 | 2.049 | 0.49 | 0.26 | 0.49 | 1.50 | N/S |
| SOX1 | 1 | 1.802 | 0.55 | 0.30 | 0.52 | 1.33 | N/S |
| ARX | 1 | 2.572 | 0.39 | 0.37 | 0.61 | 1.04 | N/S |
| LMO1 | 1 | 4.728 | 0.21 | 0.44 | 0.67 | 0.84 | N/S |
| BCL11B | 1 | 3.257 | 0.31 | 0.58 | 0.75 | 0.57 | N/S |
| POU3F3 | 1 | 4.017 | 0.25 | 0.68 | 0.83 | 0.39 | N/S |
| SHH | 1 | 5.019 | 0.20 | 0.73 | 0.89 | 0.31 | N/S |
| PITX2 | 1 | 5.806 | 0.17 | 0.80 | 0.90 | 0.22 | N/S |
